# Drought Stress Effects and Olive Tree Acclimation under a Changing Climate

**DOI:** 10.3390/plants8070232

**Published:** 2019-07-17

**Authors:** Cátia Brito, Lia-Tânia Dinis, José Moutinho-Pereira, Carlos M. Correia

**Affiliations:** CITAB—Centre for the Research and Technology of Agro-Environmental and Biological Sciences, University of Trás-os-Montes and Alto Douro, 5000-801 Vila Real, Portugal

**Keywords:** climate change, drought, *Olea europaea*, recovery, tolerance mechanisms

## Abstract

Increasing consciousness regarding the nutritional value of olive oil has enhanced the demand for this product and, consequently, the expansion of olive tree cultivation. Although it is considered a highly resilient and tolerant crop to several abiotic stresses, olive growing areas are usually affected by adverse environmental factors, namely, water scarcity, heat and high irradiance, and are especially vulnerable to climate change. In this context, it is imperative to improve agronomic strategies to offset the loss of productivity and possible changes in fruit and oil quality. To develop more efficient and precise measures, it is important to look for new insights concerning response mechanisms to drought stress. In this review, we provided an overview of the global status of olive tree ecology and relevance, as well the influence of environmental abiotic stresses in olive cultivation. Finally, we explored and analysed the deleterious effects caused by drought (e.g., water status and photosynthetic performance impairment, oxidative stress and imbalance in plant nutrition), the most critical stressor to agricultural crops in the Mediterranean region, and the main olive tree responses to withstand this stressor.

## 1. Introduction 

The olive tree (*Olea europaea* L.) is one of the emblematic crops of the Mediterranean region, where most of the world’s olive oil is produced [1]. Olive oil is widely known as the main source of fat in the so-called Mediterranean diet, being related to several beneficial effects on human health, due to its balanced fatty acid composition and antioxidant properties. Therefore, the consumption and demand for olive oil is increasing all over the world [1]. The Mediterranean region is characterized by severe summer conditions, including low rainfall, excessive heat load and high daily irradiance. Among the constituents of summer stress, drought is usually the most critical, although it is highly exacerbated by the others. Moreover, the Mediterranean region is a particularly susceptible area to climate change, a major challenge for agriculture [2]. According to the Intergovernmental Panel on Climate Change (IPCC) [2], climate change scenarios predict that the temperature will rise and precipitation patterns will shift, leading to higher evaporative demand and decreased soil water availability. Moreover, night-time temperatures will increase to a greater extent than daytime temperatures and the frequency and severity of drought and heat wave spells are likely to increase as well. These environmental factors cause adverse pleiotropic effects on plants’ growth and development. Specifically, a water deficit has negative repercussions on water relations, nutrient uptake, carbon assimilation, canopy dimension, oxidative pathways, phenology and reproduction processes [1,2,3,4,5,6,7,8,9] and, thus, affect crop yield and quality [10,11,12]. Meanwhile, some of these plant responses to adverse conditions are connected with defence adaptation strategies. Although the olive tree is a well-adapted species against drought [4,5,13,14,15,16], considerable energy resources will be used in these protective processes, compromising plant growth and productivity [16].

In the predicted scenarios of climate change, the risks for the olive sector will increase, particularly under rainfed conditions, which may jeopardize its economic viability. This may lead to the abandonment of traditional groves, with devastating socioeconomic (e.g., income and employment reduction in marginal regions) and environmental (e.g., soil erosion, increased risk of wildfires, changes in wildlife communities) consequences. On the other hand, the carbon sequestered by olive tree orchards (biomass + soil) could surpass the emissions created from farming operations and oil manufacturing, with the carbon footprint value (calculated for the production and sale of olive oil) being very low or even negative [17]. This outcome claims worldwide importance in that olive tree plantations may have significant CO_2_ sinks and mitigate greenhouse gas emissions caused by farming activities [17]. Thus, it is important to act to make this crop more sustainable, productive and resilient under severe adverse conditions, which are likely to be exacerbated in the Mediterranean region. Understanding how olive trees respond to drought stress is the first step to improving its profitability, allowing the selection of more resistant cultivars and identification of tolerant characteristics useful in breeding programs and in genetic engineering, as well as the development of accurate adaptation strategies according to necessities. In this context, the implications of climate change projections and an overview of the optimum conditions for olive cultivation are provided. In the following, the impact of drought on the morphological, physiological and biochemical traits, as well the acclimatisation responses of the olive tree to this stressor are critically discussed.

## 2. Olive Tree Growth Conditions and Distribution

The olive tree, belonging to the botanical family Oleaceae and genus *Olea* [18], is one of the oldest cultivated plants native of the Mediterranean Basin [19]. Ever since, it has contributed to the economy, health, nutrition, culture and sustainability of this region. Although the Mediterranean region remains the main area of cultivation, nowadays, this area extends to southern Africa, South and North America, Australia, Japan and China [1]. Olive cultivation worldwide is limited by edapho-climatic factors of Mediterranean isoclimatic zones lying between the 30th and 45th parallels on the northern and southern hemispheres [20]. The Mediterranean climate is typically mild and wet during the winter and hot and dry during the summer [21], being the Mediterranean area, it is usually also exposed to high daily irradiances, including UV radiation. Temperature is the most significant environmental factor that limits olive growing areas, while water availability is the most significant factor that limits olive yield. 

Proper olive cultivation areas have a mean annual temperature of 15–20 °C, with a minimum of 4 °C and a maximum of 40 °C [18]. Usually, the optimum temperature for olive vegetative growth ranges between 10 °C and 30 °C, while carbohydrate synthesis occurs at higher rates at temperatures ranging from 20 °C to 30 °C [22]. Olive trees require a period of low temperatures (0–7 °C) for flowering bud differentiation [18]. On the other hand, temperatures constantly above 16 °C prevent bud differentiation [22]. However, the minimum temperature should not drop below −7 °C, which can seriously damage trees, and if the temperature reaches −12 °C, can kill them. High altitudes (>800 m) are not appropriate for olive cultivation, due to the incidences of frost and the short vegetative period in those locals [18].

Despite being able to grow well even in poor, dry, calcareous and gravelly soils, the best conditions for olive tree annual bearing are deep, sandy-loam adequately supplied with nitrogen, phosphorus, potassium and water [18], while the optimal pH values range between 5.5 and 8.5 [23]. Although in some cases, olive trees can grow with a rainfall of 200 mm year^−1^ [24], it should be above 400 mm year^−1^, and values of 600 mm year^−1^, 800 mm year^−1^ and 1000 mm year^−1^ are considered sufficient, moderate and good, respectively [22]. Still, 500 mm year^−1^ is the lower limit for commercial olive yields under rainfed conditions [25].

Under low levels of photosynthetic photon flux density (PPFD), the percentage of flower bud induction and differentiation falls, and the same occurs with net photosynthetic rate (A). For the majority of the olive leaves, the ideal PPFD, depending on the genotype, must be in the range between 600 and 1000 μmol m^−2^ s^−l^, the light saturation point. On the other hand, PPFD of olive leaves must be above 20–30 μmol m^−2^ s^−l^, the light compensation point, to obtain higher assimilation rates than respiration rates [25].

The growing awareness of the nutritional value of olive oil has led to the expansion of olive tree cultivation [1]. The total estimated global land-use area for cultivating olive trees was over 10.6 and 10.8 million ha in 2016 and 2017, respectively. In both years, Spain was the country with a higher total harvesting area, followed by Tunisia, Italy, Morocco and Greece [26]. 

Globally, olive production was 20,344,597 and 20,872,788 tons in 2016 and 2017, respectively. Spain was 1st place, followed by Greece, Italy, Turkey and Morocco [26]. About 90% of the world’s olives production is for oil extraction and the remaining 10% for table olives [27]. Almost 92% of the world’s olive oil production comes from the Mediterranean region, with European Union countries (i.e., Spain, Italy, France, Greece and Portugal) responsible for 67% of global production [1]. To increase production, large areas were irrigated and fertilized, trees were adjusted to mechanical pruning and harvesting, and new orchards were planted in high and super-high-density plots [19].

## 3. Implication of the Change in Environmental Conditions for the Olive Tree

The impacts of recent extreme climate-related events, such as heat waves, droughts, floods, and wildfires, have revealed the significant vulnerability and exposure of some ecosystems to current climate variability [2]. However, while the above records are concerned, the forecasted scenarios may be worse, accounting with global temperatures rising, with special prominence at night-time, and increase in extreme events intensity and frequency [2]. Due to the uniqueness of its geographic location—in a transition zone between the arid climate of North Africa and the temperate and rainy climate of central Europe—the Mediterranean Basin is particularly vulnerable to present and future climate variability and climate change [23]. According to the Fifth Assessment Report (AR5) of the Intergovernmental Panel on Climate Change [2], the projections for the Mediterranean region also show warming in all seasons, especially in summer. Precipitation is not projected to change or will be moderately reduced in the winter half year (October to March), while it will be markedly reduced in the summer half year (April to September). The length, frequency and/or intensity of warm spells or heat waves are very likely to increase throughout the region.

The effects of weather and climate in agriculture can be felt at different levels, as changes in CO_2_ atmospheric concentration, temperature and water resource availability, among other factors, affect plants’ development and productivity, the possibility to execute agricultural operations and the geographical distribution of crops.

It is assumed that the CO_2_ assimilation rate and olive yield will decrease substantially in the context of climate change [28]. Regarding the increase in atmospheric CO_2_ concentration, a crop model projected an increase in the potential assimilation rate, and thus, in the overall productivity, even if reduced water availability controls and limits this tendency [29]. In fact, it is known that stomatal conductance (g_s_) decreases with increasing CO_2_ levels, while photosynthesis increases, leading to greater water use efficiency of several Mediterranean species, including the olive tree [30]. On the other hand, greater CO_2_ concentrations will promote weed growth, and therefore, competition with olive crops, although the nature of the damaging effects depends on the weed species [18]. An increase in other atmospheric pollutants is also expected, such as tropospheric ozone [16], whose increase has already been shown to cause reductions of g_s_ and A in olive trees [31]. In addition, the co-occurrence of other stressing factors (temperature increase and consequent increase in evapotranspiration and water demand; the decrease in water availability and the increase in saline water use for irrigation) will overcome the influence of increased atmospheric CO_2_ on A [30]. 

The expected increases in spring and autumn temperatures will prolong the growing season [32]. If there is adequate water availability during these periods, a general anticipation of a flowering date, of 1 to 2 weeks, could be expected [33]. On the other hand, very high temperatures could be catastrophic to flowering quality and, therefore, they could contribute to a lower olive yield [18]. In fact, it has been observed that trees exposed to insufficient chilling temperatures and high temperature events can flower, but the flowers are of low quality and have a low set percentage. This phenomenon has been documented in olive growing areas at low latitudes, where some olive varieties produce deformed floral buds and fruit [34]. Higher temperatures and evapotranspiration also accelerate fruit ripening, claiming the necessity to harvest early, probably at a lower maturity index than used today [35].

Warmer conditions will determine a possible north range expansion of cropping activities into regions where lower temperature was a limitation in the past [2]. Conversely, a reduction in the southerly cropping areas and crop yields will be expected. Indeed, potentially cultivable areas for olive cultivation are expected to extend northward and to higher altitudes [36,37], increasing by 25% in 50 years [37]. These shifts are expected due to the enlargement of the growing season in winter and because some southern areas will not satisfy the minimum chilling requirements [36]. The low temperature role in releasing dormancy of potentially reproductive olive tree buds was demonstrated in several studies [38,39,40], highlighting that 7.2 °C was sufficient to complete chilling requirements, while 12.5 °C provided both chilling requirement fulfilment and adequate temperature for subsequent floral bud growth and differentiation. Moreover, areas along the Atlantic shores may become viable very quickly, due to the increasingly milder winters [41]. For all these reasons, olive trees may be considered as one of the best bioindicators of climate evolution in the Mediterranean Basin [41].

Warming will also boost pest and disease generations [18]. In particular, warming will affect olive fly infestation levels across the Mediterranean Basin, altering olive production and decreasing the profitability of small olive farms in many marginal areas of Europe and elsewhere in the basin [42].

## 4. Drought Effects in Plants Morphological, Physiological and Biochemical Mechanisms

Drought is considered the most limiting factor for agricultural productivity worldwide [43]. In plants, water deficits occur when there is not enough water to absorb in order to replace water losses by evapotranspiration, or when having difficulty absorbing water (e.g., saline soils, low temperature, flooding). Consequently, plants then have a lower amount of water than it contains when in a state of maximum hydration, triggering a variety of physiological and biochemical responses at the cellular and organism levels. Thus, this section discusses in detail how water deficits affect plants, including the olive tree, their morphological characteristics and physiological and biochemical mechanisms, in order to further understand the specific olive tree mechanisms to deal with drought imposition.

### 4.1. Influence on Water Status, Growth and Plant Morphology

With drought imposition, as plant water content decreases, the cells shrink, and the cell wall relaxes, resulting in loss of turgor [44], causing a reduction in leaf water potential (Ψ) and in cell division and expansion [8]. If a water deficit is imposed early in the development, the inhibition of cell expansion results in a reduced leaf area, while if it is imposed after a substantial leaf area has developed, leaves will senesce and can fall off [44]. The number of leaves can also be affected, associated with a decrease in the number of branches and growth rate [43,44]. These responses limit the photosynthetic area, and thus, contribute to the decline in whole-canopy photosynthesis [43].

During soil drying and/or when evaporative demand is high, leaf transpiration often exceeds water transport capacity. Thus, xylem water potential decreases, increasing the susceptibility to cavitation—the aspiration of air into the transpiration stream. Cavitation events can cause embolism when the air fills the entire conduit, blocking water transport, reducing the number of functional conduits and increasing hydraulic resistance [16].

### 4.2. Influence on Stomatal and Mesophyll Conductance, Photosynthesis, Respiration and Water Use Efficiency

One of the primary drought consequences is the regulation of the stomatal aperture to restrict water losses [45]. The olive tree presents a tight control of stomatal behaviour to maintain Ψ within an adequate level, avoiding critical values and keeping them in a safe range to avoid embolism [15,46]. Stomatal regulation is influenced by both hydraulic and hormonal signals (abscisic acid, ABA), but may also vary under increasing drought and recovery conditions [46]. The actual role of each component in the stomatal control mechanism is not fully understood [45]. Hernandez-Santana et al. [45] found that in olive trees, the variable most strongly related to g_s_ response to water deficit is the leaf hydraulic conductance (K_leaf_), which decreases exponentially with leaf water potential. Moreover, K_leaf_ starts to decline immediately with dehydration, while the drop in g_s_ began only after a substantial K_leaf_ loss, suggesting a protective role of the stomata for K_leaf_ maintenance. Torres-Ruiz et al. [46] also recognized that g_s_ decline in olive trees during water deficit was more related to the loss of hydraulic functioning at the most distal organs of the plant (i.e., roots and leaves) than to the increase of ABA levels on leaves, stems and roots. Still, ABA’s effect on stomatal regulation should not be put aside, as it might promote stomatal closure in a dual way, by a direct biochemical action on guard cells and by an indirect hydraulic action through a decrease in leaf water permeability within leaf vascular tissues [47].

As stomatal aperture decreases, the entering of CO_2_ into the mesophyll also decreases, with negative consequences on A [6,48]. Moreover, the mesophyll compactness increases under drought conditions to restrict water diffusion, what also restricts the supply of CO_2_ to the carboxylation sites [49]. Thus, both diffusional limitations, i.e., stomatal (g_s_) and mesophyll (g_m_) conductance, contribute to A decline, showing a close relationship with each other [50,51]. Nonetheless, diffusional limitations to photosynthesis were not exclusively associated with leaf anatomical traits. In fact, especially in harsh environmental conditions, changes in leaf biomechanical and biochemical traits can lead to a reduction in mesophyll conductance to CO_2_ [16,50], e.g., carbonic anhydrase (CA) and aquaporins (AQPs) [51]. Carbonic anhydrase is a zinc metalloenzyme that catalyses the reversible hydration of CO_2_ [52], being involved in the maintenance of the equilibrium between CO_2_ and HCO_3_^−^ [53]. Thus, CA may facilitate the diffusion of CO_2_ through the chloroplast membrane and, therefore, the CO_2_ conductance within the chloroplast [49,50]. The relationship between the increase of water deficit severity and the reduction in CA expression and g_m_ in olive leaves [51] suggests that g_m_ can be enzymatically regulated by the CA. Furthermore, Gillon and Yakir [54] demonstrated that CA-mediated diffusion takes special importance in sclerophyllous species, such as the olive tree, which offers more resistance to CO_2_ in their cell walls, being necessary to offset this limitation by optimizing the chloroplast conductance. There is also other evidence supporting the relationship between g_m_ and AQPs, since some of them are involved in CO_2_ transport [51,55]. Additionally, AQPs can also be involved in g_s_ regulation [51,55] via their effects on water transport [56].

In response to moderately stressful conditions, g_s_ and g_m_ are the main limitations to A [16,57], but at severe stress levels the biochemical component of photosynthesis can also be inhibited [14,48,58]. Meanwhile, limitations in net CO_2_ assimilation may lead to an overexcitation and subsequent photo-inhibitory damage of photosystem II (PSII), as demonstrated in several studies by the reduction of relevant photochemical traits, as the photosynthetic electron transport rate (ETR), effective quantum efficiency of photosystem II (ΦPSII), maximal quantum efficiency of photosystem II (F_v_/F_m_), capture efficiency of excitation energy by open PSII reaction centres (F’_v_/F’_m_) and photochemical quenching (qP) and by the increase of non-photochemical quenching (NPQ) [4,9,48,59,60]. In line with the detrimental effect of drought on the photochemical processes of olive leaves, a reduced abundance of several proteins related to the photosynthetic Calvin–Benson cycle was also observed (mainly Rubisco downregulation) [61].

**Figure 1:**
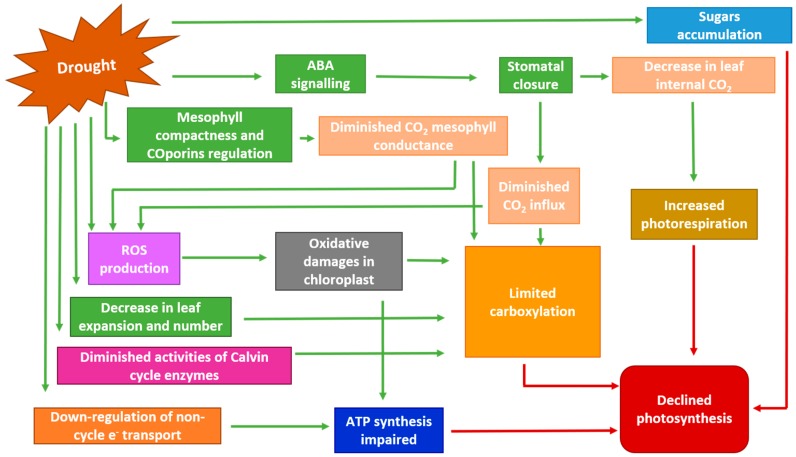
Possible mechanisms that lead to a decrease in photosynthesis under drought. ABA: abscisic acid; ROS: reactive oxygen species; ATP: adenosine triphosphate. Adapted from Farooq et al. [43].

Under conditions of drought and high light stress, the decrease in CO_2_ assimilation is usually also associated with the increase in the photorespiration rate, owing to the nature of ribulose-1,5-bisphosphate carboxylase/oxygenase (Rubisco) [43]. Nonetheless, under severe drought, photorespiration is involved in energy dissipation, reducing photoinhibition [62]. Severe drought can also cause the impairment of Rubisco and other photosynthetic enzymes [62,63], as well as the degradation of photosynthetic pigments [3,48,57]. A summary of the possible mechanisms that lead to a decrease in photosynthesis under drought is shown in Figure 1.

Although unexpected as there is no opportunity for carbon gain [64], a substantial night-time stomatal conductance (g_night_) and leaf transpiration (E_night_) were observed in a wide range of species from different functional groups and ecosystems [65,66,67], including the olive tree [7]. This generalized response suggests potential benefits related to the continued water loss during the night. For instance, it was proposed that it may improve nutrient uptake [7,65], reduce foliar temperature, limit the consumption of storage products through respiration (R) [7], and maintain the sap flux for O_2_ delivery for internal sapwood respiration and/or stem corticular photosynthesis [68]. Although the implication of overnight water loss in physiological processes remains unclear [66,67], the occurrence of this phenomenon affects the plant water balance and water-use efficiency [67], which may be detrimental under limited water conditions.

Respiration and A are strongly coupled and intrinsically interdependent because A provides photosynthetic substrates to R, and R supplies adenosine triphosphate (ATP) and carbon skeletons to sustain the requirements of plant energy processes [69]. However, in response to drought, the R trend is still not clear. Studies demonstrated that R varies, generally from inhibition, with low to moderate stress (due to the decrease of energy demand for plant growth and the impairment of some enzymes involved in R), to stimulation, with severe stress (due to the changes in metabolism to extra repair costs to offset serious damage) [70,71]. On the other hand, it was reported that olive trees are able to maintain reduced R rates during moderate to severe drought stress (relative water content of 64.7%) [7]. By reducing the metabolism, this species is able to conserve photosynthates, important for plant regrowth. Additionally, because phloem transport depends on turgor, the decrease in water potential in the phloem under severe drought may inhibit the amount of assimilates exported [44], and sugars accumulation in leaves may control photosynthesis by feedback processes [72].

Derived from changes in A and g_s_, the intrinsic water-use efficiency (WUE_i_), defined as A/g_s_, will eventually be affected by water availability. Under mild to moderate drought, WUE_i_ typically increases [6], while it may decrease under severe drought conditions, as illustrated in olive trees by Bacelar et al. [4]. However, improving WUE_i_ may not necessarily result in improving whole plant water-use efficiency (WUE_WP_) [73] and the whole plant carbon and biomass acquisition per amount of transpired water [74]. The difference in time-scale of both processes (from seconds to months) and non-accounted energy expenses in growth and maintenance in long-term water use can justify such differences [75]. Effectively, Bacelar et al. [5] reported an absence of a significant association between WUE_i_ and WUE_WP_ in different cultivars of olive trees under drought conditions.

### 4.3. Influence on Minerals Uptake and Allocation

Drought stress affects uptake, transport, and subsequent distribution of minerals within the plant [8], causing an imbalance in plant nutrition. This disequilibrium may cause important perturbations on physiological functions and on biomass accumulation [6,7], since minerals serve numerous functions in plants, as structural components in macromolecules, co-factors in enzymatic reactions, osmotic solutes and in the maintenance of charge balance in cellular compartments [76].

It is well accepted that reduced water availability results in limited nutrient uptake [77] since (i) drought reduces the whole-plant transpiration rate, due to the inferior stomatal conductance and total leaf area [7]; (ii) drought declines the soil water potential, slowing the diffusion rate of nutrients between the soil matrix and the root surface [8]; (iii) drought reduces the nutrient supply through mineralization [78]; (iv) drought impairs the activity of enzymes involved in nutrient assimilation, disturbing nutrient acquisition [40] and changing membrane permeability [77]; and (v) drought decreases the concentration of root nutrient-uptake proteins [79]. Although a lower tissue concentration of minerals is a regular response [8,77], this is not always necessarily true, as the concentration effect due to the lower production of biomass can be traduced in higher concentrations of nutrients. However, as reported previously with young olive trees, drought stress did not affect the uptake, transport, allocation and physiological use efficiency of all minerals to the same extent [80], suggesting an adaptive mechanism for growth under water limitation. For instance, drought increased both the concentration and the allocation of Mg and B to stems and roots, respectively, and increased the allocation of N to roots, at the expenses of leaves, and of P to leaves, at the expenses of stems. Phosphorus use efficiency was also improved by drought, suggesting a better distribution of phosphorus resources among the different metabolic processes involved in biomass production.

### 4.4. Influence on Redox Status

Drought increases the generation of reactive oxygen species (ROS) due to the accumulation of excess energy, which increases the photo-oxidative effect [6,61]. Usually, under mild and moderate water deficits, drought tolerant plants enhance the concentration of enzymatic and non-enzymatic antioxidants [4,63]. On the other hand, under severe drought stress, an imbalance between ROS production and the antioxidant defence system may occur [43], damaging lipids, proteins, carbohydrates, pigments and deoxyribonucleic acid (DNA) [3,4,8,9,59], which also results in increased cellular membrane damage and electrolyte leakage [7,61].

### 4.5. Influence on Hormonal Dynamics

Stress conditions often stimulate changes in the production, distribution or signal transduction of phytohormones. In fact, through the action of these molecules, plants respond to the adverse conditions modifying their physiology and biochemistry [81]. Usually considered the main stress hormone, ABA biosynthesis and accumulation is stimulated by drought, in association to a key role in g_s_ regulation, but a water deficit also influences other stress signalling pathways [47]. Thus, ABA is unequally distributed within and between the different plant organs [6,7,82,83]. Under drought stress, ethylene (ET), another important stress hormone, might inhibit plant growth [84], is involved in leaf abscission, and thus, in the reduction of water loss [85], induces remobilization of minerals from the leaves [84]; recent evidence also suggests that it might increase the accumulation of compatible solutes and reduce oxidative stress damage [86]. Moreover, it was suggested that under stress, ABA and ET act antagonistically [87]. Ethylene prevents ABA accumulation and inhibits ABA-induced stomatal closure [88] and, on the other hand, higher concentrations of ABA prevent excess ET production, which allows for shoot and root growth under drought conditions [89].

Compared to the ABA and ET responses to drought stress, no consensus has yet been achieved concerning the responses of the growth hormones auxins (Aux) and gibberellins (GA). Nevertheless, there is increasing evidence that a reduction in the levels of GA or signalling contribute to plant growth restriction, once GA inhibits the accumulation of growth suppressors, namely, DELLA (aspartic acid–glutamic acid–leucine–leucine–alanine) proteins [81]. On the other hand, the Aux responses to drought are usually contradictory, varying from decreases [90,91] to increases [92,93]. Meanwhile, Wang et al. [94] reported a marked decrease in Aux with a transient increase during the initial stage of drought adaptation. Furthermore, some studies have shown that drought influences the local Aux concentration and distribution by changes in Aux transport, allowing to maintain a balance between vegetative growth and survival [95,96]. In fact, it was well demonstrated that the Aux was unequally distributed within and among the different plant organs [6,82,83]. In addition, it was suggested that Aux could act as a stress hormone, directly or indirectly, once it was observed that several auxin-responsive genes operate during stress signalling [97]. However, hormone action cannot be considered in isolation, as the cross-talk among the different plant hormones results in synergistic or antagonistic interactions that play crucial roles in the response of plants to abiotic stress [98].

## 5. Drought Influence on Olive Crop Yield and Quality

In general, a water deficit has a negative effect on yield, fruit dry mass and oil accumulation [10,11,12,99,100], while it accelerates fruit maturation [100,101]. Regarding fruit and olive oil phenolics composition, dissimilar responses may occur. However, the general trend is the parallel increase with drought intensity [10,11,99,100,101,102,103], but oils are occasionally characterized as excessively bitter [10]. Nevertheless, from a certain threshold, the increase in quality, conferred by phenolic compounds, is not compensated by the losses in quantity, with deficit irrigation being the better option [10,103]. In line, a deficit irrigation strategy has been shown to be effective in producing quality Extra Virgin Olive Oil, by increasing the content of total phenols and sensory quality [104,105]. Meanwhile, the water deficit influence on qualitative olive oil indexes and fatty acid composition are inconsistent in the literature. Generally, the minor effects of irrigation were felt in peroxide value, free acidity and specific absorption coefficients (K_232_, K_270_, ΔK) [10,99,103]. Regarding the fatty acids profile, while in some studies no significant influence was detected [11,99], in other works, the growing season highly determined the responses [10,103], making it difficult to establish a pattern. Although there is not a direct effect from a water deficit, the severity and frequency of frosts may be higher with reduced soil and air moisture. This may be a critical event for olive quality, as harvests usually occur when early frosts start coming. Some studies have already reported that frost events damage olive fruits and, consequently, the quality of the extracted olive oil, including a decrease in pigments and phenolic compound concentrations [106,107,108,109].

## 6. Olive Tree Strategies to Withstand Drought

Plants respond to adapt and survive under limited moisture supply by inducing different strategies, which can be divided into three distinct mechanisms: drought escape, which involves a shortened life cycle or growing season, allowing plants to reproduce before the environment becomes dry; drought avoidance, which involves the adoption of mechanisms that reduce water loss from plants; and drought tolerance, which is defined as the ability to grow, flower and display economic yield under sub-optimal water supply [8].

The capacity of olive to grow under harsh conditions is due to the development of certain morphological, anatomical, physiological and biochemical responses [16], benefiting from the memory effects caused by stress pre-exposure [60]. However, these mechanisms are activated at considerable expenses to the plant in terms of energy, which causes a decrease in current-season production and compromises vegetative development, impairing next year’s production. Olive trees can slow the onset of stress (avoidance) with the ability to extract water from the soil and restricting water losses to the atmosphere. Moreover, tolerance is the ultimate drought strategy, displayed by the ability to sustain a large internal water deficit and maintain enough metabolic activity for survival [19]. However, as argued by Chen et al. [110], drought adaptability integrates much more than the drought resistance concept (i.e., drought escape, drought avoidance and drought tolerance), recovery capacity also plays a fundamental role in plants’ growth and survival. This takes special importance in Mediterranean-type ecosystems, where plants are continuously exposed to repeated cycles of drought re-watering during their life. Nevertheless, compared to development during drought, the study of recovery has been neglected. Although drought is considered the primary stressor, others such as heat and high irradiance, especially in association with each other, also impair plant functions and, therefore, different adaptive mechanisms are adopted by plants.

Olive leaves are small, with high mesophyll compactness, grouped along sclereids in spongy parenchyma and two/three palisade layers associated with the upper epidermis [13,111], being the lignin accumulation [61], the thickness and density especially marked under adverse conditions [48,56,111,112]. This particular structure reduces the internal conductance to water vapor transport [113] and provides a greater resistance to physical damage driven by desiccation [114]. Olive leaves also present a thick cuticle that prevents water diffusion through the cuticular layer. In fact, cuticular conductance is negligible when compared with g_s_, meaning that most of the transpiration is associated to the stomata [19]. Moreover, leaf surface, especially the abaxial surface, is covered with a waxy layer and stellar peltate trichomes hiding the small and abundant stomata [13], which usually increases under drought conditions [7]. These structures increase water-use efficiency, by increasing leaf boundary-layer resistance, and allowing leaves to take advantage of light rain or water condensation [115].

The stomata of olive leaves are small and present only on the abaxial surface (hypostomatous), being even smaller and denser in water shortage situations, allowing better control of water loss by transpiration [48,111]. Moreover, an efficient control of the stomatal aperture helps to maintain xylem water potential values above the safety threshold for loss of hydraulic conductance [15,16]. Although strong evidence shows that g_s_ decreases as plant Ψ becomes more negative [48,56,112], under severe conditions, stomatal control over transpiration may be not enough to prevent the loss of hydraulic conductance [112]. For some plant species, the permanent wilting point is reached when Ψ = −1.5 MPa [116], but since olive tissues can withstand very negative values of Ψ [117], the wilting point for olive ranges approximately between −2.5 MPa [118] and −3.5 MPa [119] or even has a huge capacity to sustain values below −8 MPa [120]. In fact, Moriana et al. [120] reported that rainfed olive trees with Ψ around −8 MPa extracted more 40 mm of water below the conventional wilting point (−1.5 MPa). To rainfed orchards in arid regions, this amount has significant importance since it represents around 10–15% of annual transpiration [121]. During recovery, olive trees typically show conservative behaviour, rapidly restoring water status, but exhibiting a slow recovery of g_s_ [6,15,51]. Torres-Ruiz et al. [46] found that neither hydraulic nor non-hydraulic factors were able to explain the delay in the full recovery of g_s_. These authors proposed two explanations, one involving the restoration of certain aquaporins activities, not affecting leaf hydraulic conductance directly, but the balance of osmolytes in the cells; and the other involving the occurrence of a metabolic limitation, as the increase in ABA in guard cells under drought induces the expression of hexokinases, which accelerates the stomatal closure. On the other hand, the hexokinases are also involved in sugar sensing and stimulation of the osmolytes balance that should be restarted after the recovery of water status. In addition, Brito et al. [6] showed that in line with a delay in g_s_ restoration, the intense ABA signal in droughted olive leaves after stress relief was stronger closer to the upper epidermis, suggesting its relocalisation after rehydration and a “memory” effect, which might enable a rapid response under drought restoration. Olive trees pre-exposed to drought also recover A faster than g_s_ after stress relief [6,60].

Olive trees show a high resistance to drought-induced embolism, essentially due to the small diameter of the xylem vessels and high density, leading to low xylem hydraulic conductivity that limit transpiration [5,15,122]. Furthermore, the olive root system grows quite parallel to the soil and the highest root density is found close to the trunk surface, being more suitable to absorb the light and intermittent rainfall, typical of its habitat, than water from deep layers [23]. Nevertheless, olive root growth and distribution depend largely on the soil conditions [19]. As rainfed olive trees need to explore larger soil volumes than irrigated trees to collect similar amounts of water, the total root system is greater in dry than in wet conditions [123]. In addition, under low water potential, olive trees also slow or even stop canopy growth, but still present some net photosynthesis, allowing the production of photo-assimilates that are particularly accumulated in the root system [58,61]. As a consequence, an increase in the root/canopy ratio is usual [6,58,61] in order to adjust the demand for transpiration and soil water uptake. Olive trees also benefit from hydraulic redistribution—the ability of deep roots to uptake water in moist soil layers to maintain transpiration during the hot dry season and to redistribute soil water through different root types, reducing the intensive drying of the upper soil layers [124].

To ensure the hydraulic conductance and the maintenance of water flow from roots to leaves, olive trees decrease the water potential of their tissues, establishing a particularly high gradient between leaves and roots [58,125]. Under drought conditions, the olive tree displays a strong capacity to osmotic adjustment (OA)—the accumulation of solutes—both in the leaves and roots [58,60,61,118,119]. This mechanism decreases the osmotic potential, creating a soil–plant water gradient, which enables the extraction of water from the soil at a water potential below the wilting point [119]. Osmotic adjustment is linked with passive and active osmotic regulation mechanisms, an increase in solute concentration resulting from symplastic water loss [119] and an accumulation or de novo synthesis of solutes within cells [126], respectively. Two major classes of solutes can lower the osmotic potential of tissues: inorganic cations and anions and organic compatible solutes, such as sugars, sugar alcohols, amino acids (notably proline), and quaternary ammonium compounds (notably glycine betaine) [126,127]. Some of the organic solutes can also protect cellular proteins, enzymes and cellular membranes and allow the metabolic machinery to continue functioning [14,126]. On the other hand, changes in cell wall elasticity can also contribute to drought adaptability, as demonstrated in different olive genotypes [14] and water regimes [3]. In these studies, it was interesting to notice that both increases and decreases in cell wall elasticity may aid survival under low water availability. In fact, more elastic cell walls can shrink more easily when subjected to stress, helping the maintenance of higher turgor pressure and protecting cell walls from rupturing [128], while more rigid cells may help to maintain lower water potential at any given volume than elastic ones, resulting in a higher gradient of water potential between the soil and the plant, thereby promoting more effective water uptake from drying soils [127].

Although the AQPs’ relevance in olive tree drought tolerance is still poorly explored, their involvement in precise water movement regulation underpin this [129]. The change in AQPs’ activity may serve to ensure that during stress, water moves to where it is required or is retained and where it is most critical [130]. Additionally, AQPs may be important in whole-plant rehydration during the recovery period, displaying also an important role in xylem conduit refilling after drought-induced embolism [131,132]. A downregulation of AQP genes, both under moderate or severe droughts, followed by an upregulation upon re-watering and then a return to normal levels were identified in olive trees [131,132]. Furthermore, AQPs’ responses can be correlated with the isohydric and anisohydric behaviour of plants, which can eventually switch from one to another [56] in response to changing environmental conditions, as reported for grapevines [133] or to fruit load, as stated for the olive tree [134].

**Figure 2:**
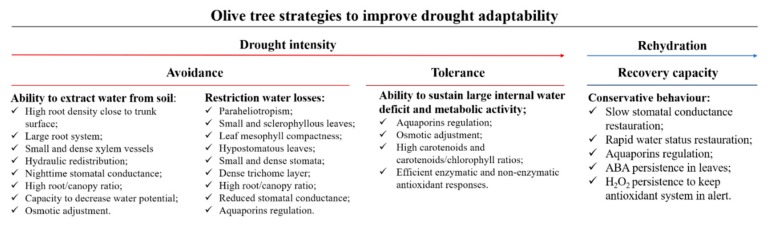
Strategies adopted by the olive tree to improve drought adaptability (i.e., drought avoidance, tolerance and recovery capacity).

Finally, the regulation of the antioxidant system is one of the most relevant mechanisms against oxidative stress caused by ROS. Reactive oxygen species play a double role in plant physiology, but whether ROS would act as signalling molecules or might cause oxidative stress to the tissues depend on the refined balance between its production and scavenging [135]. The increase in carotenoids and the carotenoids/chlorophylls ratio is considered one of the mechanisms developed by the olive tree to protect the photosynthetic apparatus against photooxidation [60]. Moreover, the increment in some antioxidant enzymes activities, such as ascorbate peroxidase, catalase, superoxide dismutase, glutathione reductase and/or in non-enzymatic antioxidant mechanisms, such as the accumulation of phenolic compounds, tocopherols, carotenoids, ascorbate and glutathione, were commonly described in olive trees under drought conditions [3,4,9,58,59,60]. On the other hand, in a study conducted by Abdallah et al. [60], it was demonstrated that upon re-watering, olive trees still exhibited higher levels of hydrogen peroxide (H_2_O_2_), a known signalling ROS, possibly to keep the antioxidative system on alert. Moreover, olive trees that were drought-primed showed an alleviation in oxidative stress in relation to plants exposed to drought for the first time [60]. A summary of the strategies adopted by the olive tree to improve drought adaptability (i.e., drought avoidance, tolerance and recovery capacity) is shown in Figure 2. 

## 7. Cultivars’ Response to Drought

Considerable genotypic differences are present among different cultivars, which employ different mechanisms to cope with drought [3,5,9,13,14,136,137]. In general, olive cultivars native to dry regions have more capability to acclimate to drought conditions than cultivars which originated in regions with a more temperate climate [14]); still, the identification of the traits of the more resistant cultivars is ambiguous, as it depends on the cultivars compared in the studies available. 

Bacelar and colleagues [3,5,13,14] studied Portuguese and Spanish cultivars, including *Cobrançosa*, *Manzanilla*, *Negrinha*, *Blanqueta*, *Arbequina*, *Madural* and *Verdeal Transmontana*. *Cobrançosa* exhibited good protection against water loss through high-density foliar tissue and by thick cuticle and trichome layers, while *Manzanilla* and *Negrinha* enhanced their sclerophyll by building parenchyma tissues and increasing protective structures like the upper cuticle and both the upper and lower epidermis [13]. Among *Cobrançosa*, *Madural* and *Verdeal Transmontana* cultivars, *Cobrançosa* had a more efficient water transport through the xylem, a more enhanced water-use efficiency of biomass production [5] and a high capability to osmotic adjustment and protection against oxidative stress [3]. *Madural* also had a thick upper epidermis, a thick palisade parenchyma, a high stomatal density, high capability for osmotic adjustment and increased tissue rigidity, but less effective mechanisms against oxidative stress [3]. Meanwhile, *Cobrançosa*, *Manzanilla* and *Negrinha* employ a prodigal water-use strategy and high tissue elasticity [14]. Moreover, *Manzanilla* accumulated high levels of proline [14]. *Arbequina* had a thinner trichome layer, implying that the leaves were less protected against water loss, but the development of smaller leaves may reduce water loss at the whole-plant level [13]. Conversely, present high levels of soluble proteins, which may represent an increased activity of oxidative stress defence enzymes [14]. *Verdeal Transmontana* did not exhibit osmotic adjustment capacity but was able to increase tissue elasticity and total soluble protein concentration [3]. *Blanqueta* had larger leaves and some anatomical traits that may lead to high water loss, especially from the adaxial surface [13]. *Arbequina* and *Blanqueta* had high tissue rigidity, employed a conservative water-use strategy, had lower photosynthetic rates and a high midday depression in photosynthesis [14].

Among Greek cultivars, *Gaidourelia* showed higher phenolic concentration and antioxidant activity and lower lipid peroxidation and photochemical damage than *Kalamon*, *Koroneiki* and *Megaritiki* [9], while in a study comparing *Konservolia*, *Zard* and *Amigdalolia* cultivars, *Konservolia* had higher chlorophyll and total carbohydrates concentrations and higher antioxidant enzymes activities, whereas the highest total phenol and proline levels were recorded in the *Zard* cultivar [136].

In another study, the effect of drought on the inhibition of growth was higher in *Fishomi* than in *Dezful*, *Amigdalolia* and *Conservolia* cultivars. *Dezful*, *Amigdalolia* and *Conservolia* were able to preserve higher levels of relative leaf water content and membrane stability index under drought stress, due to the higher concentrations of soluble carbohydrates, proline, potassium and calcium in their leaves [137].

## 8. Concluding Remarks and Future Challenges

Future climatic conditions in the Mediterranean region will certainly cause harmful consequences to olive tree physiology and yield, particularly under rainfed conditions. Although the olive tree has several mechanisms that allow for good acclimation to drought, they are activated at the expense of carbon reserves and may be detrimental with the increased duration and intensity of the stress. This consequence may lead to the abandonment of traditional olive groves and desertification in extensive areas, with devastating economic, social and environmental costs. Moreover, young plantations require some irrigation for their establishment due to their poorly developed root system and reduced capacity for storing water, that will be traduced in a great amount of water consumption. Thus, understanding the main effects and the response mechanisms adopted by the olive tree to cope with drought is crucial to improving crop management strategies and to designing more sustainable and productive crop systems and saving water resources. This review consolidated knowledge on how the olive tree responds to drought, but also demonstrated that much more effort is needed to fully understand these responses and their implication from the plant to the ecosystem level in a changing world. For instance, night-time stomatal conductance and sap flux, different classes of aquaporins activities, leaf hydraulic conductance, mesophyll conductance, the role of phytohormones cross-talk and other signalling molecules, such as H_2_O_2_, and recovery processes are topics less explored in olive tree response to environmental stressors. Moreover, higher investment in integrated approaches with proteome and the corresponding physiological responses can profoundly change our understanding of olive tree adaptability.

## Abbreviations and Symbols

A	net photosynthetic rate
A/g_s_	intrinsic water use efficiency
ABA	abscisic acid
AQPs	aquaporins
ATP	adenosine triphosphate
Aux	auxins
CA	carbonic anhydrase
CO2	carbon dioxide
DNA	deoxyribonucleic acid
E_night_	night-time transpiration
ET	ethylene
ETR	photosynthetic electron transport rate
F’_v_/F’_m_	capture efficiency of excitation energy by open PSII reaction centres
F_v_/F_m_	maximal quantum efficiency of photosystem II
GA	gibberellins
g_m_	mesophyll conductance
g_night_	night-time stomatal conductance
g_s_	stomatal conductance
H_2_O_2_	hydrogen peroxide
HCO_3_^−^	bicarbonate anion
K_232:_	excitation coefficient at 232 nm
K_270_	excitation coefficient at 270 nm
K_leaf_	leaf hydraulic conductance
NPQ	non-photochemical quenching
O_2_	oxygen
OA	osmotic adjustment
PPFD	photosynthetic photon flux density
PSII	photosystem II a
qP	photochemical quenching
R	respiration
ROS	reactive oxygen species
WUE_i_	intrinsic water use efficiency
WUE_WP_	whole plant water use efficiency
ΔK	variation of the specific extinction
ΦPSII	effective quantum efficiency of photosystem II
Ψ	leaf water potential
